# Supplementary material for: Polyketide synthase gene clusters in the ericoid mycorrhizal fungus Oidiodendron maius: genome-wide prediction, expression profiling, and functional characterization of knockout mutants
Source: Front Plant Sci. 2026 Sep 16;17:1924430. doi: 10.3389/fpls.2026.1924430 (PMC13623903; doi:10.3389/fpls.2026.1924430)
Supplement: Supplementary file 1 [file SupplementaryFile1.zip › Supplementary Materials/Supplementary Folder 1.zip!/Oidma1_AssemblyScaffolds_Repeatmasked/knownclusterblast/cluster32/jgi.p_Oidma1_20513_mibig_hits.html]

| MIBiG Protein | Description | MIBiG Cluster | MiBiG Product | % ID | % Coverage | BLAST Score | E-value |
| --- | --- | --- | --- | --- | --- | --- | --- |
| ALV82389.1 | alcohol\_dehydrogenase | BGC0001370 | Lipopeptide\_8D1-1\_/\_lipopeptide\_8D1-2\_biosynthetic\_gene\_cluster | 37 | 96.3068181818 | 237 | 1.9e-62 |
| ADM34147.1 | dehydrogenase | BGC0001084 | Notoamide\_/\_stephacidin\_biosynthetic\_gene\_cluster | 30 | 98.2954545455 | 168 | 1.9e-41 |
| CAE22477.1 | putative\_dehydrogenase | BGC0000720 | Tobramycin\_biosynthetic\_gene\_cluster | 30 | 84.6590909091 | 111 | 1.6e-24 |
| CAH18550.1 | putative\_cyclitol\_1-dehydrogenase,\_TobE | BGC0000721 | Tobramycin\_biosynthetic\_gene\_cluster | 30 | 84.6590909091 | 111 | 1.6e-24 |
| ABI91459.1 | Alcohol\_dehydrogenase,\_zinc-binding\_domain\_protein | BGC0001094 | Enacyloxin\_biosynthetic\_gene\_cluster | 22 | 90.3409090909 | 56 | 6e-08 |
